# Functional Analysis of Starch Metabolism in Plants

**DOI:** 10.3390/plants9091152

**Published:** 2020-09-06

**Authors:** Yong-Gu Cho, Kwon-Kyoo Kang

**Affiliations:** 1 Department of Crop Science, Chungbuk National University, Cheongju 28644, Korea; 2 Division of Horticultural Biotechnology, Hankyong National University, Anseong 17579, Korea

**Keywords:** storage carbohydrate, starch biosynthesis, structural genes, transcription factors, post-translational modification, starch mutants

## Abstract

In plants, starch is synthesized in leaves during the day-time from fixed carbon through photosynthesis and is mobilized at night to support continued respiration, sucrose export, and growth in the dark. The main crops where starch is biosynthesized and stored are corn, rice, wheat, and potatoes, and they are mainly used as food resources for humankind. There are many genes that are involved in starch biosynthesis from cytosol to storage organs in plants. ADP-glucose, UDP- glucose, and glucose-6-phosphate are synthesized catalyzed by UDP-invertase, AGPase, hexokinase, and P- hexose-isomerase in cytosol. Starch composed of amylopectin and amylose is synthesized by starch synthase, granule bound starch synthase, starch-branching enzyme, debranching enzyme, and pullulanase, which is primarily responsible for starch production in storage organs. Recently, it has been uncovered that structural genes are controlled by proteins derived from other genes such as transcription factors. To obtain more precise information on starch metabolism, the functions of genes and transcription factors need to be studied to understand their roles and functions in starch biosynthesis in plants. However, the roles of genes related to starch biosynthesis are not yet clearly understood. The papers of this special issue contain reviews and research articles on these topics and will be a useful resource for researchers involved in the quality improvement of starch storage crops.

## 1. Introduction

Starch is the one of three major nutrients for human life, which is the main storage carbohydrate in plants and is used for food and various other purposes. The major crops where starch is biosynthesized and stored are corn, rice, wheat, and potatoes, and are mainly used as food resources for humankind. Recently, starch has also attracted attention as a source of bioethanol and various industrial new materials. Starch is synthesized in leaves during the day-time from fixed carbon through photosynthesis and is mobilized at night to support continued respiration, sucrose export, and growth in the dark [1]. The starch of grains is made up of about 20% amylose and about 80% amylopectin, and the physicochemical properties of starch vary depending on the ratio of amylose to amylopectin [2]. It is a natural polymer material in which glucose produced by photosynthesis of plants is polymerized and mainly stored in grains, tubers, and roots. Its importance was proven by starch deficiency mutants that do not grow well or even die under short day conditions [3]. In storage organs such as potato tubers or developing seeds, starch acts as a long-term carbon reservoir and is later rebuilt in the development stage to support the reproductive growth stage [3]. The quality improvements of starch in many crops, like potato, rice, wheat, and maize will contribute greatly to food security.

Starch synthesis begins with the production of ADP glucose, a substrate for starch synthase. The production of ADP glucose in the photosynthetic chloroplasts of plant leaves occurs in the Calvin– Benson cycle by converting fructose-6 phosphate to glucose-1 phosphate (Glc-6-P) through glucose-6-phosphate (Glc-6-P). ADP-glucose pyrophosphorylase catalyzes the generation of Glc-1-P and ATP to ADP glucose and pyrophosphate (PPi) [1].

There are many genes that are involved in starch biosynthesis from cytosol to storage organs in plants. ADP-glucose, UDP-glucose, and glucose-6-phosphate are synthesized catalyzed by UDP- invertase, AGPase, hexokinase, and P-hexoseisomerase in cytosol. Starch is synthesized by starch synthase, granule bound starch synthase, starch-branching enzyme, debranching enzyme, and pullulanase, which is primarily responsible for starch production in storage organs [2]. Recent studies on the regulation of starch biosynthesis are evolving in a wide range of environmental and metabolic signaling processes. In plastid, AGPases that are regulated by transcription factors, *agpS1-2* and *agpL1-3* [4], catalyze the conversion of Glc-1-P to ADP glucose and pyrophosphate (PPi). Then, starch synthases including *SSs*, *GBSSI*, *BE*, *DBE*, *ISA*, and *PUL*, use ADPglc to add glucose moieties to the amylose and amylopectin molecules. This enzyme is known to be controlled by the transcription factors, *OsSERF1*, *OsbZIP58*, and *OsRPBF* in rice, and *ZmEREB156*, *ZmbZIP91*, and *ZmNAC36* in maize [4].

However, our knowledge of the signal transduction cascade is not yet complete. In particular, there is a shortage of knowledge on the molecular identity of the sensor, intracellular signaling pathways, and integration between photosynthetic and metabolic signals. Research over the past few years has also expanded the understanding of the role of post-translational protein modification and protein–protein interactions in the regulation of starch synthesis [5]. Evidence is emerging that starch synthesis is controlled by reversible protein phosphorylation and protein complex formation.

Scientists have studied mainly the structural genes that are related to specific biosynthetic pathways and they have tried to link their functions one by one in an attempt to understand the whole biosynthesis pathway in plants. Recently, it has been uncovered that structural genes are controlled by proteins derived from other genes such as transcription factors [4]. To obtain more precise information on starch metabolism, the functions of genes and transcription factors need to be studied to understand their roles and functions in the starch biosynthesis in plants. For analyzing the function of target genes, forward and reverse genetics approaches are useful. However, it is not yet clear whether these mechanisms are important in vivo and whether their role can be generalized to other plant species. More research will be needed to increase the understanding of the important aspects of starch synthesis control and apply the knowledge to crop improvement.

The advent of genome-editing tools has enabled researchers to modify specific genomic sequences. A gain-of-function mutation (also called activating mutations) alters a gene product so that its effect is stronger (enhanced activation) or replaced by another abnormal function [6,7]. A loss-of-function mutation, also known as inactivating mutations, causes a gene product to have little or no function [8]. However, there are still many genes and transcription factors that are not yet clearly understood with respect to their role and function. This special issue includes genes in starch biosynthesis, transcriptional regulation, post-translational protein modification, and many functional genes isolated from gain-of-function and loss-of-function.

## 2. Highlight of Special Issues

### 2.1. Key Genes in Starch Biosynthesis

ADP-glucose is synthesized from Glc1P and ATP by ADP-glucose pyrophosphorylase (AGPase) and produced as a heterotetramer (L_2_S_2_) composed of two large subunits and two small subunits. The AGPase small subunit serves as a catalytic function, whereas the AGPase large subunit primarily serves to regulate the allosteric regulatory properties of AGPase [1,9]. Amylose, the linear form of starch, is produced primarily through the activity of granular bound starch synthase (GBSS). Amylopectin synthesis relies on coordinated interactions between more than 17 different genes encoding isoforms of starch synthase, starch branching enzyme, isoamylase, pullulanase, and phospholase1. Among them, starch synthase I plays a key role in extending the short chain from the A or B1 chain of amylopectin to DP 8 to 12 in the degree of polymerization (DP) of 6 to 7 chains at the branch point [10,11]. Starch synthase II plays a distinct role in catalyzing the formation of the intermediate chain of amylopectin (typically DP 13–25) [12]. SSIII mainly catalyzes the synthesis of amylopectin B2 to B4 chain, some of its functions overlap with that of SSII in amylopectin biosynthesis [13,14]. SSIV plays a critical role in the priming of starch granule formation, the shape of starch granules, and the degree of starch accumulation. In addition, depending on the plant species, the function may be partially supported by SSIII [15,16]. SBEI primarily promotes the production of longer chains (B1–B3), while SBEIIa and SBEIIb primarily enhance the generation of short amylopectin chains (DP 6–12) and affect the structure and phenotype of amylopectin during starch biosynthesis [17,18]. The ISAI homomultimer or ISAI/ISAII heteromultimers has a higher affinity for relatively long external branches and has a greater effect on the amylopectin structure, whereas ISAII recognizes special branch points and promotes the ability of ISAI to degenerate. It can be indirectly involved in a branch and remove nearby branches [19,20,21]. In addition, ISAIII plays an important role in starch decomposition by partially complementing the function of ISAI/ISAII heteromultimers, debranching of short outer chains of glucans, and affecting the activities of α- amylase and β-amylase [22,23]. PUL has a function that partially overlaps with ISA and is involved in cleaving short branching chains in the process of starch biosynthesis [24,25].

### 2.2. Transcriptional Regulation Network

Due to the production of ADP-glucose being the first committed step in the starch pathway, much research has been carried out on the transcriptional regulation of AGPase. AGPase is a heterotetramer that contains two large and two slightly smaller subunits [26]. The large subunits have more regulatory roles, while the small subunits have both catalytic and regulatory properties [27]. Some transcription factors (TFs) such as *SUSIBA2* [28], *SRF1* [29], *OsRSR1* & *OsbZIP58* [30,31], and *ZmaNAC36* & *ZmbZIP91* [32,33], are involved in the regulation of the expression of several AGPase isogenes in plants. A coordinated regulation of gene expression in the source and sink organs is largely regulated by sugar status. Little is known about the detection and signaling mechanisms that mediate these processes. Barley endosperm under development includes *SUSIBA2*, a WRKY transcription factor that is involved in source-sink communication and sucrose-mediated control of starch synthesis [28]. Additionally, ethylene signaling has recently been linked in the transcriptional control of starch synthesis in rice, involving the ethylene receptor *ETRC* [34] and the *AP2/EREBP* family transcription factor [30]. Regulation of the transcription of one protein can affect the abundance of another protein. This may be the case, for example, in the case of the rice mutant Floury Endosperm2 (FLO2), which multiplied the expression of many starch genes [35].

In a transcription network, some genes are regulated by some proteins, while others are structural and therefore more important than others. Many genes metabolize, but do not control DNA transcription or RNA translation on their own. Thus, there are many different types of genes in the transcription network, such as regulatory genes and endpoint genes [4].

### 2.3. Post-Translational Protein Modification

Post-translational modification (PTM) of a protein allows rapid regulation of protein function in response to metabolic and environmental changes. Phosphorylation of proteins is known to play an important role in regulating the distribution of light energy in photosynthesis. ADP-glucose pyrophosphorylase (AGPase) was revealed to be a target of post-translational redox regulation, including the reversible formation of an intermolecular Cys bridge between Cys-82 of two small subunits of the heterotetrameric enzyme [3,36]. Reduction of AGPase results in a dramatic change in the kinetic properties of the enzyme, increasing substrate affinity and sensitivity to the allosteric activator 3PGA, while reducing the sensitivity of inhibition by Pi [36]. Recently, a unique type of NADP-dependent thioredoxin reductase C (NTRC) was reported to be included in the post- translational redox regulation of AGPase [37]. In addition, many enzymes involved in starch degradation have been reported to be redox regulated, which may mean coordinated regulation of starch synthesis and degradation by redox signals [3]. Recent researches have suggested that reversible protein phosphorylation plays an important role in the regulation of starch metabolism. More study is needed to investigate the in vivo relevance of this mechanism [38]. With regard to this point of view, the possible interactions between redox regulation and protein phosphorylation are also interesting methods to follow [39].

In some aspects, research points to the importance of post-translational modifications to regulate starch metabolism. That is, given that mRNA levels are not directly related to protein levels, it is necessary to study how plants regulate mRNA expression in the early stages of transcription and how proteins are post-translationally modified and used for starch biosynthesis.

Mutants are useful tools for analyzing the function of a target gene; for this reason, large quantities of mutants and other research data have been produced by physical mutagenesis (i.e., irradiation), chemical mutagenesis, or genetic methods [40]. Most of the functional loss variants do not have useful agricultural traits, are generally recessive, and are difficult to use directly for plant breeding.

Recently, there have been more chances to improve our understanding through advanced scientific tools such as GWAS, MABc, gene pyramiding, overexpression, knock-out, RNAi, and gene editing. This Special Issue of Plants will highlight the identification, function, roles, and relationships of genes and transcription factors in starch biosynthesis, and their interactions through the processes in the starch synthesis pathway of plants.
